# Directional and Fast Photoluminescence from CsPbI_3_ Nanocrystals Coupled to Dielectric Circular Bragg Gratings

**DOI:** 10.3390/mi12040422

**Published:** 2021-04-13

**Authors:** Yan Hua, Yuming Wei, Bo Chen, Zhuojun Liu, Zhe He, Zeyu Xing, Shunfa Liu, Peinian Huang, Yan Chen, Yunan Gao, Jin Liu

**Affiliations:** 1 State Key Laboratory of Optoelectronic Materials and Technologies, School of Physics, Sun Yat-sen University, Guangzhou 510275, China; huay7@mail2.sysu.edu.cn (Y.H.); weiym8@mail.sysu.edu.cn (Y.W.); chenb255@mail.sysu.edu.cn (B.C.); liuzhj23@mail2.sysu.edu.cn (Z.L.); hezh26@mail2.sysu.edu.cn (Z.H.); xingzeyu1996@hotmail.com (Z.X.); liushf7@mail2.sysu.edu.cn (S.L.); huangpn3@mail2.sysu.edu.cn (P.H.); 2 College of Interdisciplinary Study, National University of Defense Technology, Changsha 410073, China; 3 State Key Laboratory for Mesoscopic Physics, Frontiers Science Center for Nano-optoelectronics, School of Physics, Peking University, Beijing 100871, China

**Keywords:** perovskite nanocrystals, photoluminescence, CBGs, coupling

## Abstract

Lead halide perovskite nanocrystals (NCs), especially the all-inorganic perovskite NCs, have drawn substantial attention for both fundamental research and device applications in recent years due to their unique optoelectronic properties. To build high-performance nanophotonic devices based on perovskite NCs, it is highly desirable to couple the NCs to photonic nanostructures for enhancing the radiative emission rate and improving the emission directionality of the NCs. In this work, we synthesized high-quality CsPbI_3_ NCs and further coupled them to dielectric circular Bragg gratings (CBGs). The efficient couplings between the perovskite NCs and the CBGs resulted in a 45.9-fold enhancement of the photoluminescence (PL) intensity and 3.2-fold acceleration of the radiative emission rate. Our work serves as an important step for building high-performance nanophotonic light emitting devices by integrating perovskite NCs with photonic nanostructures.

## 1. Introduction

All-inorganic perovskite nanocrystals (NCs) are an emerging class of material for lighting and display technologies due to their excellent optoelectronic properties. Compared with organic–inorganic hybrid perovskite, all-inorganic perovskite NCs exhibit several advantages, including wider wavelength tenability [1], higher quantum yield at ambient temperature [2], and lower cost. They are easy to synthesize and can be integrated with various photonic nanostructures [3,4,5]. The all-inorganic perovskite have found wide applications in optoelectronic devices, such as nanolasers [6,7,8,9,10,11], LED [12,13], solar cells [14,15,16,17], and photodetectors [18,19]. Notably, recent studies also suggest that the perovskite NCs are excellent emitters of non-classic light at cryogenic temperatures [20,21,22]. To further improve the brightness and the coherence properties of the single-photon sources based on perovskite NCs, it is highly desirable to couple the perovskite NCs to photonic nanostructures. In this work, we synthesize high-quality CsPbI_3_ NCs emitting at visible light (See Appendix A) and further efficiently couple the NCs to the SiN circular Bragg gratings (CBGs) to simultaneously enhance their radiative rate and improve their emission directionality. Further developments in this direction may result in high-performance integrated devices with applications in nanophotonics and quantum optics.

## 2. Experimental Section

**Figure 1:**
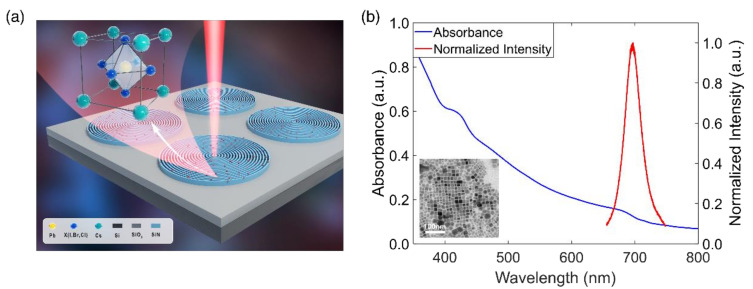
(**a**) A schematic of the devices consisting of Si_3_N_4_ circular Bragg gratings (CBGs) covered by a layer of polymethyl methacrylate (PMMA) mixed with perovskite NCs. Inset: the atomic structure of the perovskite nanocrystals (NCs). (**b**) The absorption and photoluminescence (PL) spectra of the NCs. Inset: transmission electron microscopy (TEM) image of perovskite NCs., the scale bar is 100 nm.

Figure 1a is an artistic sketch of the proposed device in which the CsPbI_3_ NCs are located on top of the CBGs. The inset is the atomic structure of the NCs, showing the perovskite structure. The absorption and the photoluminescence (PL) spectra of the CsPbI_3_ perovskite NCs at room temperature is shown in Figure 1b. Our perovskite NCs feature a wide absorption range above 700 nm. The inhomogeneous spectral broadening of NCs is around 50 nm, which is caused by size difference of the individual NCs. We characterized the morphology and size distribution of CsPbI_3_ NCs by using transmission electron microscopy (TEM), as shown in the inset of Figure 1b. An average size of 20 nm was obtained, which led to a PL peak of around 700 nm at room temperature.

**Figure 2:**
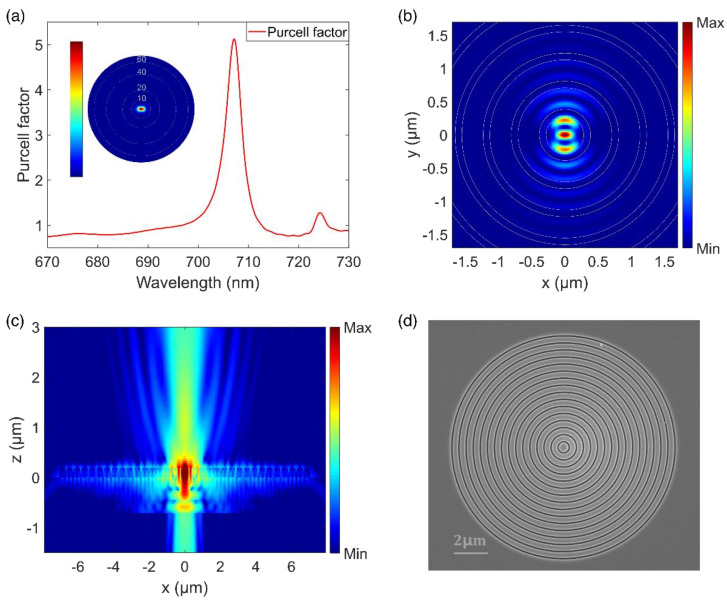
(**a**) The simulated Purcell factor as a function of the wavelength. Inset: the far-field polar plots at the cavity resonance of 707 nm. (**b**) Electric field intensity distribution of CBG cavity mode in the X–Y plane. (**c**) Electric field intensity distribution of CBG cavity mode in the X–Z plane. (**d**) The top-view SEM image of the CBG.

Targeting an emission wavelength of 700 nm, we designed and optimized the CBGs via the finite difference time domain (FDTD) method (See Appendix A for details). The simulated device consists of a silicon substrate, a 710 nm thick SiO_2_ layer (*n* = 1.45) and a 196 nm thick Si_3_N_4_ (*n* = 2.02) CBG. The center disk radius, grating period and trench width of the CBGs are 300, 423 and 101 nm, respectively. As presented in Figure 2a, a Purcell factor of ~5.5 was obtained around 707 nm and the resonance wavelength could be fine-tuned in our experiment by varying the size of the central disk of the CBG. The inset is the far-field polar-plot at the cavity resonance, showing that most of the emitted photons were highly directional, i.e., within a divergent angle of 10 degrees. Figure 2b,c shows the electric field intensity distributions of the cavity mode in the X–Y plane and the X–Z plane, respectively. The intensity distribution in the X-Y plane is confined in the center of CBG, suggesting the lateral dissipation is largely suppressed. The numerical simulation of the intensity in the X–Z plane shows clearly that a substantial amount of photons were emitted upwards with a narrow divergent angle. The scanning electron microscope (SEM) image of the fabricated CBGs is presented in Figure 2d. The sizes of critical features in the fabricated devices are very close to our designed parameters.

**Figure 3:**
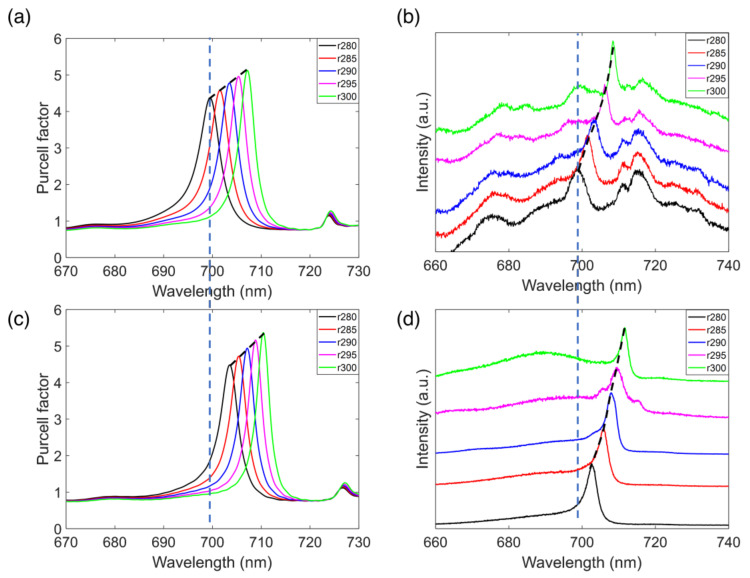
(**a**) Simulation of the cavity modes of the CBGs with different radiuses of the central disk before spin-coating the perovskite NCs. (**b**) Measured PL spectra of the cavity modes of the CBGs with different radiuses of the central disk before spin-coating the perovskite NCs. (**c**) Simulation of the cavity modes of the CBGs with different radiuses of the central disk after spin-coating the perovskite NCs. (**d**) PL spectra of the cavity modes of the CBGs with different radiuses of the central disk after spin-coating the PMMA and perovskite NCs.

In our experiment, we fabricated a set of CBGs with the central disk radius varying from 280 to 300 nm at steps of 5 nm (See Appendix A for fabrication details). The cavity modes of the CBGs can be probed by the intrinsic PL from the Si_3_N_4_ under the excitation of a 532 nm laser. Figure 3a shows the cavity modes of CBGs with different radiuses through FDTD simulation before coating the polymethyl methacrylate (PMMA) layer, and Figure 3b shows the measured spectra of the cavity modes. By comparing Figure 3a,b, we can see that our simulations and experimental results match well. The cavity modes shifted from 698 to 708 nm with the increase of the size of the central disk, which is indicated by the dashed line. Figure 3c presents the simulation of the cavity modes with the encapsulation of a PMMA layer, which shows a 3–4 nm red-shift of the cavity modes. In the experiment, the NCs containing a solvent consisting of a mixture of 0.5% PMMA and CsPbI_3_ solution (with a volume ratio of 1:2) were subsequently spin-coated onto the CBGs (See Appendix A). More details of the solvent are discussed in the experimental section. As shown in Figure 3d, the experimental results were in very good agreement with the simulation, showing a 3–4 nm red shift of the cavity modes by the PMMA layer. The spectra in Figure 3d were collected by a single mode fiber as a spatial filter, which gave rise to a better signal-to-noise ratio of the cavity modes (details provided in Appendix A).

**Figure 4:**
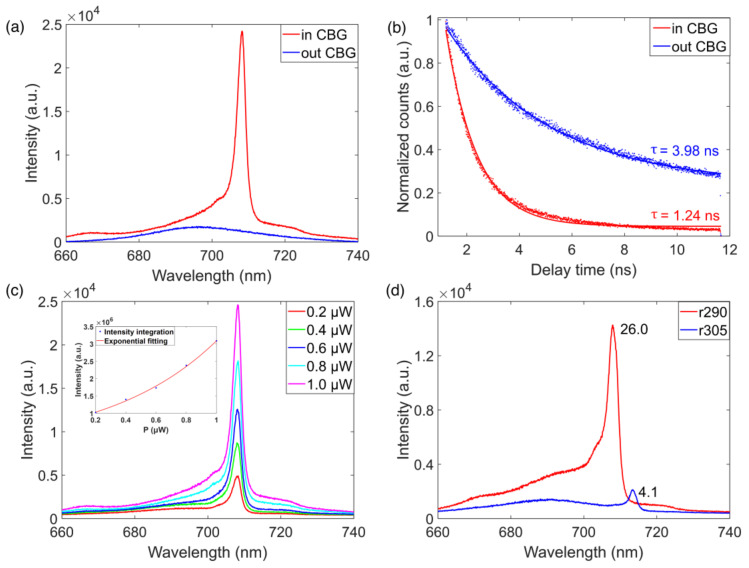
(**a**) PL spectrum of perovskite NCs in the center of CBG disk (red) and outside the structure (blue). (**b**) The time-resolved PL from perovskite NCs in the center of CBG (red) and outside the structure (blue). The dots are experimental data and solid lines are numerical fits. (**c**) PL spectra of the coupled device at different pumping intensities. (**d**) PL spectra of perovskite NCs for the resonant and detuned conditions.

To investigate the coupling between the perovskite NCs and the CBGs, we compared the PL intensities from the perovskite quantum dots (PQDs) spatially outside and inside the CBG. As shown in Figure 4a, the PL intensity of the NCs in the CBG denoted by the red curve was 45.9 times stronger than that of NCs outside the CBG denoted by the blue curve. Such a PL intensity enhancement is mostly due the coupling the NCs to the CBG cavity modes, which is efficiently collected by the optical objective. To quantitatively study the coupling strength, the lifetimes of the spontaneous emission from the CsPbI_3_ PQDs were measured, as shown in Figure 4b. A 532 nm pulsed laser with an intensity of 0.6 μJ/cm^2^ was used to excite the NCs (See Appendix A for Optical Measurement details). For the NCs without the CBG, a lifetime of 3.98 ns was extracted from the decay curve, shown in blue. Once coupled to the CBG, a significantly shortened lifetime of 1.24 ns was obtained, as shown by the red decay curve, which resulted in a Purcell factor of 3.2. Both the intensity enhancement and the lifetime reduction in our experiment strongly suggest that efficient couplings between the NCs and the CBGs were achieved.

We further studied the power-dependent PL for the coupled PQDs. Figure 4c shows representative spectra of the NCs coupled to the CBG under different excitation powers. When increasing the pump intensity from 0.2 to 1.0 μW, the PL intensity increased accordingly but no lasing signature was observed, as shown in the inset of Figure 4c. To further confirm that the enhanced PL was caused by the coupling between the NCs and CBGs, the CBG modes were deliberately detuned from NCs emission. The PL enhancement for the resonant case was 26, which is significantly higher than that of 4.1 for the off-resonant case, as presented in Figure 4d.

## 3. Conclusions

In conclusion, we have synthesized CsPbI_3_ NCs emitting at 700 nm. By depositing NCs encapsulated in a PMMA layer on top of CBGs, a 45.9-fold enhancement of PL intensity and a 3.2-fold reduction of the lifetime were obtained, suggesting that efficient coupling between the NCs and the cavity modes of the CBGs was achieved. Further developments in this direction, e.g., coupling single NCs to the CBG, may result in coherent single-photon sources with low fabrication costs. Our work presents a viable path for building active nanophotonic devices based on perovskite NCs.

## Appendix A

### Appendix A.1. Numerical Simulation

We used a 3D finite difference time domain (FDTD) simulation to design the cavity resonance of the CBG by changing the radius of the CBG central disk. The simulated device is made of a 196 nm thick Si_3_N_4_ (*n* = 2.02) layer, a 710 nm thick SiO_2_ (*n* = 1.45) layer and a silicon substrate. The period of the grating is 423 nm and the trench width of the CBG is 101 nm. We set the perovskite nanocrystals as an electric dipole source placed on the center of CBG, covering the wavelengths from 670 to 730 nm. When the central disk radius was 300 nm, a resonance peak was observed at 707 nm with a maximum Purcell factor of ~5.5. We added PMMA (*n* = 1.47) in the gaps of the CBG. The thickness of PMMA is around 60 nm. We also calculated the collection efficiency of the CBG varying the central disk radius.

### Appendix A.2. Synthesis of the Perovskite NCs

For the preparation of the Cs-oleate precursor, a mixture of Cs_2_CO_3_ (0.407 g), oleic acid (OA, 1.25 mL) and octadecene (ODE, 20 mL) was loaded into a 50 mL three-neck flask and dried under vacuum for 1 h at 120 °C. Then the reaction temperature was raised to 150 °C under nitrogen for 2 h to completely dissolve the Cs_2_CO_3_. After being cooled to room temperature, the Cs-precursor was kept in a glovebox. As Cs-oleate would precipitate out of ODE at room temperature, it needs be pre-heated to 100 °C before injection. For the synthesis of CsPbI_3_ NCs, 0.376 mmol PbI_2_ and ODE (10 mL) were loaded into a 50 mL three-neck flask and dried under vacuum for 1 h at 120 °C, followed by the injection of oleic acid (OA, 1 mL) and oleylamine (OAm, 1 mL) under a nitrogen atmosphere. The solution temperature was elevated to 170 °C. A total of 0.8 ml of Cs-oleate, pre-heated to 100 °C, was quickly injected into this reaction mixture. After 5 s, the reaction flask was immediately cooled to room temperature with an ice bath. Finally, the CsPbI_3_ NCs were purified by centrifugation (8000 rpm for 10 min) and the precipitate was dispersed in toluene to form a stable solution.

### Appendix A.3. Fabrication of the CBGs

The basic structure of the CBGs consisted of a central circular disk surrounded by periodic circular Bragg gratings. The circular Bragg gratings were designed to meet the second-order Bragg conditions. They can reduce the lateral leakage of photons effectively and increase the vertical light extraction. The CBGs were made from a 196 nm SiN layer that was grown on a SiO_2_ layer by plasma-enhanced chemical vapor deposition (Oxford Plasma Pro System100 ICP180-CVD). To fabricate the designed structure, the sample was spin-coated with electron-beam resist (ARP-6200). The patterns were defined onto the resist by using electron-beam lithography (Raith Vistec EBPG5000+ 100 kV). These patterns were transferred onto the SiN layer by reactive ion etching (RIE). The resist was eventually removed by RIE using gentle oxygen plasma.

### Appendix A.4. Coupling Device Preparation

In order to improve the stability of the perovskite quantum dots, we mixed 0.5% polymethyl methacrylate (PMMA) with the CsPbI_3_ solution with a volume ratio of 1:2. The mixed solution was spin-coated on the CBGs sample at a speed of 8000 rpm for 2 min. The sample was then thermally annealed at 60 °C for 1 min.

### Appendix A.5. Optical Measurement

The device was characterized using a home-built PL setup. We measured the cavity modes by shining a continuous wave (CW) laser at 532 nm on the CBG center. The optical power was around 30 μW and was focused with a 50× objective (Olympus, NA = 0.90). The PL spectra were collected by a spectrometer with a 300 lines mm^−1^ grating (Princeton Instrument SP2758) and detected by the integrated charge-coupled devices (CCD). The residual laser was filtered out using a 540 nm long-pass filter. The exposure time was set to be 5 s. The same setup was used to characterize the CsPbI_3_-coated CBGs. For time-resolved decay measurement, the sample was excited with a pulsed laser at repetition rates of 86 MHz (532 nm, 5 ps). PL was detected with an avalanche photon detector that was connected to a single-photon counting module (PicoHarp 300).
